# Time to cessation of exclusive breastfeeding and associated factors among women with children aged 6–24 months in Kilimanjaro region, northern Tanzania: A community-based cross-sectional study

**DOI:** 10.1371/journal.pone.0259041

**Published:** 2021-10-28

**Authors:** Farida Ali, Sia E. Msuya, Redempta Mamseri, Melina Mgongo, Innocent B. Mboya

**Affiliations:** 1 Institute of Public Health, Department of Epidemiology and Biostatistics, Kilimanjaro Christian Medical University College (KCMUCo), Moshi, Tanzania; 2 Better Health for African Mother and Child (BHAMC), Moshi, Tanzania; 3 Institute of Public Health, Department of Community Medicine, Kilimanjaro Christian Medical University College (KCMUCo), Moshi, Tanzania; 4 Department of Community Medicine, Kilimanjaro Christian Medical Center (KCMC), Moshi, Tanzania; 5 School of Mathematics, Statistics & Computer Science, University of KwaZulu Natal, Pietermaritzburg, Scottsville, South Africa; University of Waterloo, CANADA

## Abstract

**Background:**

Exclusive breastfeeding (EBF) up to six months is sub-optimal globally. Tanzania has surpassed the World Health Assembly (WHA) target of increasing the rate of exclusive breastfeeding among children below six months to at least 50% by the year 2025 the median age of cessation of EBF is only three months.

**Objective:**

To determine the time to cessation of exclusive breastfeeding and its associated factors among women with children aged 6–24 months in Kilimanjaro region, Northern Tanzania.

**Methods:**

This was a secondary analysis of data from a community-based cross-sectional study conducted between April 2016 and April 2017 in Kilimanjaro region, northern Tanzania. In the parent study, a multistage sampling technique was used to select study participants and interviewed using a questionnaire. Data for 1291 mother-child pairs were analyzed using STATA version 15. Kaplan-Meier method with the log-rank test estimated and compared the survivor functions across covariate levels. Cox regression proportional hazards models estimated the hazard ratios (HR) and their 95% confidence intervals (CI) for factors associated with time to cessation of exclusive breastfeeding.

**Results:**

The prevalence of cessation of exclusive breastfeeding before six months was 68.7%, with a median age of cessation of four months (95% CI: 3, 4). In comparison to women living in Siha district, women living in Moshi Municipal (HR = 1.61; 95% CI = 1.24, 2.09), Same (HR = 1.32; 95% CI = 1.06, 1.65) and Mwanga (HR = 1.53; 95% CI = 1.20, 1.96) districts, had higher hazards of cessation of exclusive breastfeeding before six months. Women who received breastfeeding counselling at antenatal care had a lower hazard to cease EBF (HR = 0.76, 95% CI 0.65, 087) compared to those who did not receive breastfeeding counselling.

**Conclusion:**

The median age of cessation of EBF is unsatisfactory but at least higher (four months) than the national level estimate of three months. District specific interventions and breastfeeding counselling at antenatal care are essential for improving time to cessation of exclusive breastfeeding. Promotion of adequate ANC visits remains one of the critical interventions to improve BF practices and other reproductive health outcomes.

## Background

Breast milk is a natural food composed of essential nutrients that are easily digestible and sufficient to provide infants with all required nutrients that are enough for producing energy and growth during the first six months of life [1]. WHO recommends optimal breastfeeding, i.e., all children to be initiated to breastfeeding within one hour after delivery, exclusively breastfed (EBF) up to six months, timely introduced to complementary feeding and continue breastfeeding up to two years or beyond [2]. The benefits of optimal breastfeeding are well documented in the literature. It includes improving child health and survival, development, intelligent quotient and reduces the risks of developing obesity in later life. EBF extend to the mother also, protects the mother from breast cancer, postpartum haemorrhage and helps in child spacing [3]. EBF is among the nutritional targets that have been set by the World Health Assembly (WHA) and other international collaborators as the key to improve global nutritional goals to reach Sustainable Development Goals (SDG) [4].

Despite the known benefits of optimal breastfeeding practices, the practice is sub-optimal, globally 58% of infants are not exclusively breastfed in the first six months [5]. In recognition of the benefits of EBF, the WHA has set a target of reaching 50% by the year 2025 and 60% by 2030 [6]. However, with the current rate of EBF practice, the global progress is still low to meet targets to 2025 [6]. In Sub Saharan Africa (SSA) where there is high disease burden, high exposure to use contaminated water, and high HIV prevalence, reports show that, about 57% of infants are not exclusively breastfeed [7]. These reports suggest that high proportion of infants are not benefiting from the intervention. Tanzania is among the countries that has surpassed the WHA goal of reaching EBF rate at least 50% [8]. Evidence from Tanzania demographic and health survey shows that there are some improvement in the median age of cessation of EBF [8]. The current data reported a median time to cessation of three months at the national level and two and a half months in the Northern zone of Tanzania [8].

Different factors have been reported to be associated with cessation of EBF. These include, mother’s age (<20 years), perceived inadequate breast milk, caesarian section delivery, not receiving infant feeding counselling at antenatal care (ANC) [9–11].

Cessation of EBF before the age of six months increases risk of child morbidity and mortality mainly due to diarrhoea and acute respiratory tract infections [12]. Suboptimal EBF also contributes to one-third of childhood undernutrition [13].

DHS reports the national levels of EBF practice and median duration of EBF, but does not assess the factors associated with the practice [8]. Also data on EBF practice at the sub national level (region and district level) where the EBF intervention are designed and implemented are missing from the DHS report [8]. Understanding time to cessation of EBF and its associated factors at the subnational levels is essential for a better planning of intervention. This study aimed to determine the time to cessation of EBF and its associated factors among women with children aged 6–24 months in the Kilimanjaro region, northern Tanzania.

## Methods

### Study design and setting

The study utilized secondary data from a community-based cross-sectional study conducted in the Kilimanjaro region, northern Tanzania in April 2016 and April 2017. The study conducted by the Institute of Public Health (IPH) of Kilimanjaro Christian Medical University College (KCMUCo) aimed to assess infant and young feeding practices among children under five years of age in the region. The study recruited mother-child pairs from six districts out of seven in the Kilimanjaro region, namely; Moshi Municipal, Rombo, Same, Mwanga, Hai, and Siha. Data for women with children 6–24 months, who reported time to cessation of exclusive breastfeeding were analyzed.

Kilimanjaro region is among the 30 administrative regions in Tanzania Mainland with an area size of 13,250 km^2^. This region is located in the Northern part of the country and has a population of approximately 1,640,000 people, 845,000 (51.6%) being females. More than three-quarters of the population resides in rural areas [14]. Agriculture and livestock keeping are the main economic activities but complemented by tourism, manufacturing, and business activities.

The region has 18 hospitals, 51 health centers, and 333 dispensaries, which are owned by the Government, and faith-based organizations, or private institutions/ individuals.

### Study population, sample size, and sampling

**Fig 1:**
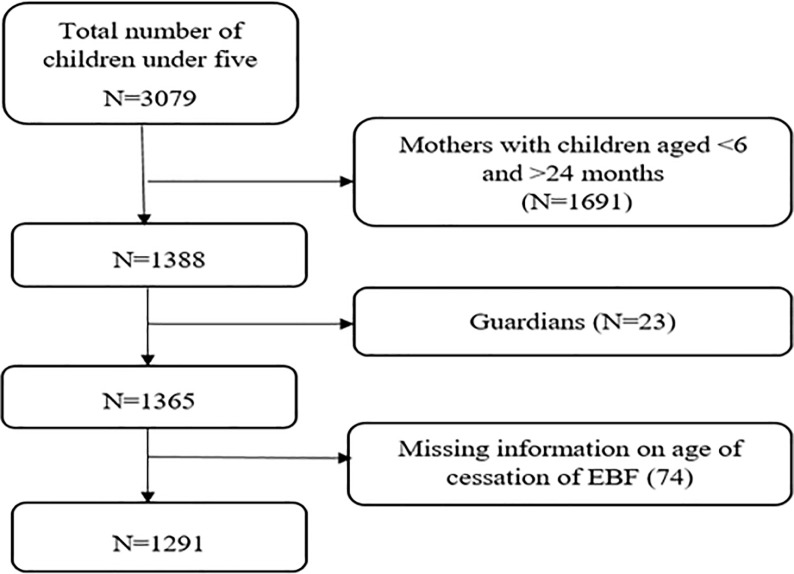
Schematic diagram showing the sample size used in the analysis.

The parent study applied a multistage sampling technique and enrolled 3079 women with children aged less than five years from each of the six districts where interviewed using structure questionnaire by trained medical students. The current study included women with children aged 6–24 months, with complete information on time to cessation of EBF. About 1691 mothers with children aged <6 months and >24 months, 23 guardians/ non biological mothers and 74 children with missing information on time when exclusive breastfeeding was ceased were not included in the analysis. Data for 1291 women were, therefore, analyzed in this study **(Fig 1)**.

### Study variables and variable measurements

The dependent variable in this study was time to cessation of EBF in months. Time to cessation of EBF was assessed using birth recall information. Participants were asked about “the age when the child was given other foods/fluids apart from breast milk”. Then, women who had ceased EBF before six months were considered to have experienced the event (EBF cessation) and “censored if otherwise”.

The independent variables included socio-demographic characteristics of the mother (age, marital status, education level, employment status, district of residence), child (sex, age and birth weight) and reproductive and health characteristics (birth order, number of ANC visit, place of birth, breastfeeding counseling during ANC visits and postnatal care and timing of breastfeeding initiation.

### Data management and analysis

Data were analyzed by using STATA version 15. Categorical variables were summarized using frequencies and percentages and continuous variables using means or median (standard deviation or interquartile range). Time to cessation of EBF was estimated using the Kaplan Meier method. Significance of survival time differences by background characteristics tested using the log-rank test. The Cox proportional hazard models estimated the hazard ratios (HR) and their 95% confidence intervals (CI) for factors associated with time to cessation of EBF controlled for confounding. All independent variables with *p-*value <0.10 in the bivariate analysis were entered in a multivariable Cox regression model to adjust for the potential confounding effect. Step-wise regression was used for model building and variable selection. The final model was selected based on the model with the lowest Akaike information criteria (AIC). Final results were presented after post-estimation diagnostics. P-value<0.05was considered to be statistically significant in the multivariable analysis.

### Ethical clearance and consent to participate

Ethical approval for the current study was sought from the Kilimanjaro Christian Medical University College Research and Ethical Review Committee. Ethical approval number PG.026/2019. Permission to use the data was sought from the Director, Institute of Public Health of the Kilimanjaro Christian Medical University College. The parent study obtained written, informed consent from study participants. Interviews were conducted in a private place around the household, and participants identified using unique identification numbers to ensure confidentiality and privacy of participant information.

## Results

### Socio-demographic characteristics of the mother

A total of 1291 women with children aged 6–24 months were included in this study. The mean age of the mothers was 28 ± 6.7 years. Majority (81.1%) were in a union, 63.5% had primary education level, and only 14.5% were employed **(Table 1)**.

**Table 1:** Background characteristics of the mother (N = 1291).

Variable	Frequency	Percentage
**Districts**		
Rombo	284	22.0
Moshi Municipal	126	9.8
Same	289	22.4
Mwanga	162	12.6
Hai	220	17.0
Siha	210	16.2
**Age in years** *	** **	
15–24	466	36.2
25–34	570	44.2
35–49	252	19.6
Mean ± SD	28±6.7	
**Marital status** *	** **	
In-union	1045	81.1
Non- union	244	18.9
**Education level** *	** **	
Non-formal	42	3.3
Primary	818	63.5
Secondary and above	429	33.2
**Employed**		
No	1104	85.5
Yes	187	14.5
**Partner education level** *		
Non-formal	33	2.9
Primary	677	59.7
Secondary and above	425	37.4

*Variables with missing values. SD: standard deviation.

### Socio-demographic characteristics of the child

More than- half of the children (50.9%) were females. Their mean age was 15 ± 5.6 months. The prevalence of low birth weight was 7.7%, **(Table 2)**.

**Table 2:** Child background characteristics (N = 1291).

Variable	Frequency	Percentage
**Sex**		
Male	634	49.1
Female	657	50.9
**Age in months**		
6–11	448	34.7
12–17	365	28.3
18–24	478	37.0
Mean ± SD	15± 5.6	
**Birth weight**		
Low	97	7.7
Normal	1170	92.3
**Birth order** *		
1	451	34.9
≥2	840	65.1

*Variables with missing values. IQR: interquartile range. SD: standard deviation.

Note: low birth weight: <2.5 Kilograms, Normal birth weight: ≥2.5 Kilograms.

### Reproductive characteristics and breastfeeding practices

More than three quarters (77.7%) of women in this study attended ≥4 ANC visits during the most recent pregnancy. Nearly two-thirds (64.7%) received breastfeeding counselling at ANC, 87.6% delivered in the health facilities, 56.3% received breastfeeding counselling during postnatal care. Among all women, 70% had early initiation of breastfeeding, and only 68.7% were ceased exclusively breastfed before the age of six months **(Table 3)**.

**Table 3:** Reproductive characteristics and breastfeeding practices (N = 1291).

Variable	Frequency	Percentage
**Birth order**		
1	451	34.9
≥2	840	65.1
**Median (IQR)** *	2 (1, 3)	
**Number of ANC visits** *		
< 4	285	22.3
≥4	996	77.7
**Breastfeeding counselling during ANC** *		
No	453	35.3
Yes	829	64.7
**Place of birth** *		
Health facility	1127	87.6
Home/Others	159	12.4
**Breastfeeding counselling during PNC** *		
No	725	56.3
Yes	564	43.7
**Breastfeeding initiation** *		
Within one hour	902	70.0
More than one hour	386	30.0
**Exclusive breastfeeding cessation** *		
<6 months	887	68.7
≥6 months	404	31.3

*Variables with missing values. ANC: antenatal care. IQR: interquartile range, PNC: postnatal care.

### Time to cessation of exclusive breastfeeding

**Fig 2:**
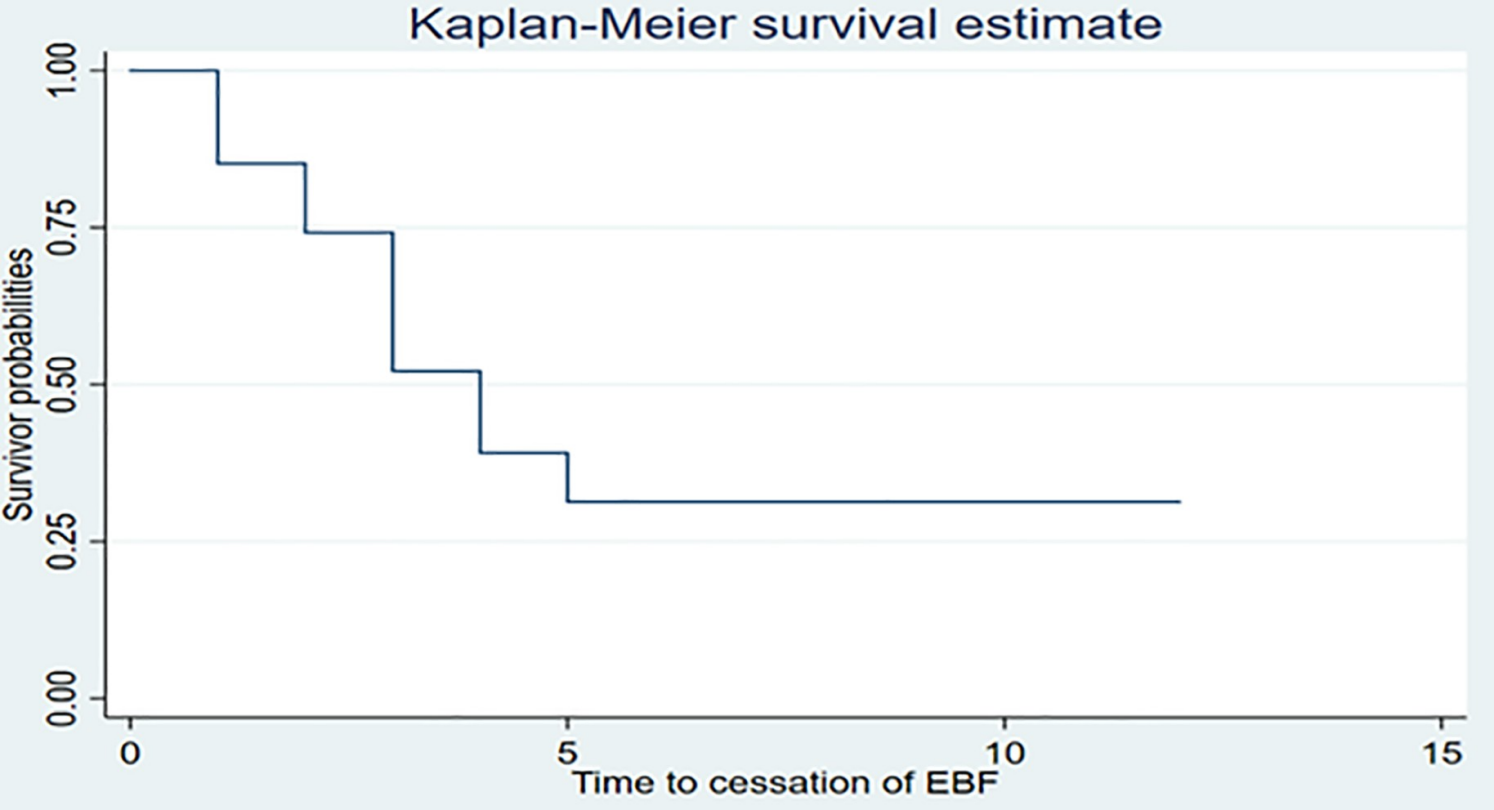
Survival function for time to cessation of exclusive breastfeeding (N = 1291).

The overall median age (months) to cessation of EBF for the Kilimanjaro region was four months (95% CI: 3, 4). EBF was ceased to almost 50% of the children by the age of four months. **(Fig 2)**.

The overall person time to cessation of EBF among 1291 mother-child pair was 50.2 person months. The rate of cessation of EBF before six months was 17.7 per 100 person months (95% CI = 16.5, 18.9). There was a significant difference in the median age of cessation of EBF between women by districts, breastfeeding counselling at ANC and during PNC and sex of the child. Rates of cessation of EBF before six months varied significantly across six districts ranging from 14.7 per 100 person months (95% CI = 12.4, 17.5) in Siha district to 23.5 per 100 person months (95% CI = 19.3, 28.6) per 100 women in Moshi Municipality. The rates were higher among women who did not receive breastfeeding counselling at ANC 21.9 per 100 person months (95% CI = 19.7, 24.3) than those who receive 15.7 per 100 person months (95% CI = 14.5, 17.1) and women who received breastfeeding counselling during PNC 19.4 per 100 person months (95% 17.8, 21.2) than those who did not receive 15.6 per 100 person months (95% CI = 14.1, 17.3) **(Table 4)**.

**Table 4:** Rate of cessation of EBF by background characteristics (N = 1291).

Variables	Events	Median age of EBF cessation (95% CI)	Rate of cessation of EBF/100 (95% CI)	P-value
**Districts**				
Rombo	182	4 (4,5)	15.0 (13.0, 17,4)	<0.001
Moshi Municipal	99	3 (3, 3)	23.5 (19.3, 28.6)	
Same	210	3 (3, 4)	20.0 (17.5, 22.9)	
Mwanga	126	3 (3,3)	23.4 (19.7, 27.9)	
Hai	138	4 (4, 5)	15.3 (12.9, 18.0)	
Siha	132	4 (4, 5)	14.7 (12.4, 17.5)	
**Maternal education level**				
Non-formal	27	4 (3, 5)	15.3 (10.5, 22.2)	0.209
Primary	578	4 (3, 4)	18.4 (17.0, 20.0)	
Secondary and above	280	4 (3, 4)	16.5 (14.6, 18.5)	
**Breastfeeding counselling at ANC**				
No	349	3 (3, 3)	21.9 (19.7, 24.3)	<0.001
Yes	533	4 (4, 4)	15.7 (14.5, 17.1)	
**Place of birth`**				
Health facility	764	4 (4, 4)	17.3 (16.1, 18.6)	0.043
Home/Others	159	3 (3, 4)	20.5 (17.1, 24.6)	
**Breastfeeding counselling during PNC **				
No	526	3 (3, 4)	19.4 (17.8, 21.2)	0.001
Yes	359	4 (4,4)	15.6 (14.1, 17.3)	
**Sex of the child**				
Male	450	3 (3, 4)	18.9 (17.2, 20.7)	0.042
Female	436	4 (4, 4)	16.6 (15.1, 18.2)	
**Birth weight of the Child kilograms**				
Low birth weight	60	4 (4, 5)	14.8 (11.5, 19.0)	0.096
Normal birth weight	805	4 (3,4)	17.7 (16.5, 19.0)	
**Breastfeeding initiation**				
Within one hour	601	4 (3, 4)	17.0 (15.7, 18.4)	0.054
More than one hour	284	4 (3, 4)	19.4 (17.2, 21.7)	

ANC: antenatal care. PNC: Postnatal care. CI: confidence interval: EBF: exclusive breastfeeding, PNC: post natal care.

### Factors associated with time to cessation of exclusive breastfeeding

In the bivariate Cox proportional hazard regression analysis, district of residence, breastfeeding counseling at ANC, and during PNC were significantly associated with time to cessation EBF at 5% significant level. Women living in Moshi Municipality (HR = 1.63; 95% CI 1.07, 2.11), Same (HR = 1.38; 95% CI 1.22, 1.72), and Mwanga (HR = 1.61; 95% CI 0.83, 2.05) districts had higher hazards of cessation of EBF compared to women living in Siha district. Women who received breastfeeding counselling at ANC (HR = 0.74; 95% CI 0.64, 0.84) and during postnatal care (HR = 0.81; 95% CI = 0.71, 0.93) had a lower hazard of cessation of EBF **(Table 5)**.

**Table 5:** Factors associated with time to cessation of EBF (N = 1291).

Variable	CHR (95% CI)	P-value	AHR (95% CI)	P-value
**Districts**				
Siha	1		1	
Rombo	1.03 (0.82, 1.29)	0.787	0.97 (0.77, 1.22)	0.795
Moshi Municipal	1.63 (1.07, 2.11)	<0.001	1.61 (1.24, 2.09)	<0.001
Same	1.38 (1.22, 1.72)	0.004	1.32 (1.06, 1.65)	0.013
Mwanga	1.61 (0.83, 2.05)	<0.001	1.53 (1.20, 1.96)	0.001
Hai	1.06 (0.82, 1.34)	0.637	1.01 (0.79, 1.23)	0.928
**Maternal education level**				
Non-formal	1			
Primary	1.17 (0.80, 1.72)	0.424	-	-
Secondary & above	1.05 (0.71, 1.57)	0.792	-	-
**Partner education level**				
Non-formal	1			
Primary	1.02 (0.66, 1.56)	0.935	-	-
Secondary & above	1.06 (0.69, 1.63)	0.798	-	-
**Number of ANC visits**				
< 4	1			
≥4	0.96 (0.82, 1.12)	0.581	-	-
**Breastfeeding counselling at ANC**				
No	1		1	
Yes	0.74 (0.64, 0.84)	<0.001	0.76 (0.65, 0.87)	<0.001
**Place of birth**				
Health facility	1		1	
Home/Others	1.19 (0.98, 1.45)	0.074	1.16 (0.95, 1.41)	0.135
**Breastfeeding counselling during PNC**				
No	1		1	
Yes	0.81 (0.71, 0.93)	0.003	0.92 (0.79, 1.06)	0.237
**Sex of the child**				
Male	1		1	
Female	0.89 (0.78, 1.01)	0.072	0.89 (0.78, 1.01)	0.073
**Birth Weight of the child**				
Low	1			
Normal	1.22 (0.94, 1.58)	0.142	-	-
**Timing of breastfeeding initiation**				
Early	0.88 (0.77, 1.02)	0.089	0.90 (0.78, 1.04)	0.150
Late	1		1	

ANC: antenatal care. CHR: crude hazard ratio. AHR: adjusted hazard ratio. CI: confidence intervals, PNC: post natal care.

In the multivariable analysis after adjusting for breastfeeding counselling during postnatal care, place of birth, child sex and breastfeeding initiation, district of residence and breastfeeding counselling at ANC were the only factors that significantly associated with time to cessation of EBF. A higher hazard of cessation of EBF was among women living in Moshi Municipal (HR = 1.61; 95% CI = 1.24, 2.09), Same (HR = 1.32; 95% CI = 1.06, 1.65) and Mwanga (HR = 1.53; 95% CI = 1.20, 1.96) compared to women in Siha. Lower hazard of cessation of EBF was among women who received counselling at ANC (HR = 0.76; 95% CI = 0.65, 0.87) compared to those who did not receive counselling during ANC **(Table 5)**.

## Discussion

This study revealed that the median age of EBF cessation was low (4 months). District of residence and receiving breastfeeding counselling at antenatal care were the only factors significantly associated with time to cessation of exclusive breastfeeding.

The median age of cessation of EBF in this study was four months, which is higher than the national estimate of 3 months [8] and that from Congo (10.9 weeks) [9]. A higher estimate in this study could be due to the fact that, the study design was a community-based cross-sectional, and information on time to cessation of EBF was based on recall since birth approach compared to the hospital-based prospective cohort design in Congo. Also, the TDHS enrolled a nationally representative sample as opposed to this study. The time to cessation of EBF in this study is lower than that reported in Ethiopia (6 months) and those in India and Sri Lanka [10, 15, 16]. The findings in Ethiopia could be because the majority of the participants were urban residents, delivered at the health facilities and initiated breastfeeding early.

District of residence was significantly associated with time to cessation of EBF. Women living in Mwanga, Same and Moshi Municipality had a higher hazard of cessation of EBF compared to those in Siha district. Differences in social, culture and belief which exists between tribes in these districts could be the possible reasons for these variations.

Breastfeeding counselling at ANC was associated with lower hazard of cessation of EBF, similar to findings in Tanzania [17] and Ethiopia [18]. The availability of trained staffs and guidelines for infant and young child feeding practices, including optimal EBF, might explain the observed association. Furthermore, the repeated sessions of health education/counselling during ANC visits enhance women understanding and preparedness. Experience sharing from women who exclusive breastfed their children previously might have influenced the findings as well. In the Kilimanjaro region, the majority of primary health care facilities provide reproductive, and child health services. About 98% of pregnant women receive ANC from skilled birth attendants [8]. The finding imply that during ANC visits it is the opportunity period for promoting breastfeeding practices including optimal EBF. This intervention together with promoting adequate ANC visits are essential to promote EBF practices.

### Strength of the study

This study has determined the time to cessation of EBF and associated factors in six districts of the Kilimanjaro region.

### Limitation of the study

The reported time to cessation of exclusive breastfeeding for some children might have been biased because the collected information was based on recall since birth approach, which has the potential for recall bias. Also, there could be social desirability bias due to women self-reporting of time to cessation of EBF.

## Conclusion and recommendations

The median age to cessation of EBF is unsatisfactory, although it was slightly higher than the national estimate. There is a need to strengthen health promotion interventions to improve EBF for up to six months. District of residence and breastfeeding counselling at ANC were the factors significantly associated with time to cessation of EBF. Strengthening breastfeeding counselling at ANC is essential. Interventions to improve EBF should be context specific. Also, more studies need to be conducted to explore the differences in factors associated with time to cessation of EBF across districts in the Kilimanjaro region.

## Supporting information

S1 Data (XLS)

## List of abbreviations

EBF: Exclusive Breastfeeding
KCMUCo: Kilimanjaro Christian Medical University College
MoHCDGEC: Ministry of Health Community Development Gender Elderly and Children
TDHS: Tanzania Demographic and Health Survey
UNICEF: United Nations Children’s Fund
WHA: World Health Assembly
